# The evolving epidemiology of acute gastroenteritis in hospitalized children in Italy

**DOI:** 10.1007/s00431-021-04210-z

**Published:** 2021-07-29

**Authors:** Brigida Stanyevic, Margherita Sepich, Samanta Biondi, Giampiero Igli Baroncelli, Diego Peroni, Maria Di Cicco

**Affiliations:** 1 School of Medicine, University of Pisa, Via Roma n. 55, 56126 Pisa, Italy; 2 Paediatrics Unit, Pisa University Hospital, Via Roma n. 67, 56126 Pisa, Italy; 3 Department of Clinical and Experimental Medicine, University of Pisa, Via Roma n. 55, 56126 Pisa, Italy

**Keywords:** Rotavirus, Diarrhoea, Adenovirus, Salmonella, Ondansetron

## Abstract

Few data are available on the prevalence and features of acute gastroenteritis (AGE) in hospitalized children in Italy, where specific rotavirus vaccines were introduced into the national vaccination plan in 2017. To evaluate vaccination effects on AGE epidemiology, we analysed data from children aged ≤ 18 years admitted for AGE at the University Hospital of Pisa in 2019, comparing them with those recorded in 2012. Demographical, clinical, diagnostic, and treatment data were collected reviewing medical records and were therefore compared. In 2019 and 2012, 86 (median age 2.5 years [IQR 1.4–5.9]) and 85 children (median age 2.3 years [IQR 1.3–5.1]) were respectively admitted with AGE. The most common symptoms were diarrhoea and vomiting; decreased skin turgor was more frequent in 2019 (54% and 34% respectively, *p* = 0.01). Viral infections were more common than bacterial ones; in 2019, a decrease in rotavirus infections (67% and 22%, *p* = 0.003) and an increase in adenovirus infections (50% and 10%, *p* = 0.002) and in the number of patients with negative stool testing (58% and 39%, *p* = 0.04) were found.

*Conclusions*: Viral infections are the leading cause of AGE in hospitalized children in Italy. The introduction of rotavirus vaccines did not reduce the number of hospitalizations per year. Adenovirus and other non-routinely screened viruses may be undergoing a selection process making them common causative agents for AGE.

**Table:** 

**What is Known:** *• Rotavirus is the leading cause of acute severe gastroenteritis in children worldwide, especially < 5 years of age.* *• The introduction of specific vaccines may be changing its epidemiology.* *• Few data are available on acute gastroenteritis in hospitalized children in Italy.*	
**What is New:** *• Viral infections are the leading cause of acute gastroenteritis in hospitalized children in Italy.* *• Specific vaccines are reducing rotavirus infections, but adenovirus and other non-routinely screened viruses may be undergoing a selection process making them common causative agents for gastroenteritis.*

**Supplementary Information:**

The online version contains supplementary material available at 10.1007/s00431-021-04210-z.

## Introduction

Acute gastroenteritis (AGE) is one of the most common infectious diseases in children and represents an important burden for public health. According to the Global Health Data Exchange in 2016, diarrhoea was the eighth and the fifth leading cause of death among all ages and under 5 years, respectively. The prevalence and mortality of AGE rise dramatically in low-income countries where health care access is limited and where safe water and sanitation are difficult to achieve [1]. In industrialized countries, the mortality rate drops significantly while morbidity still represents a challenge with a noticeable number of hospitalizations, outpatient evaluations, and related costs [2–4]. Patients with AGE typically show stools with decreased consistency (loose or liquid) and/or an increase in the frequency of evacuations (typically ≥ 3 in 24 h), with or without fever or vomiting, usually lasting less than a week and never more than 2 weeks [5]. Severe or prolonged symptoms may require hospitalization and microbiological investigations [4, 5]. Viruses account for 70–90% of cases of AGE [6, 7], with rotavirus infection being the leading cause of severe AGE and death, mainly in children under 5 [1]. In Europe, in 2012 the annual incidence of community-acquired rotavirus AGE among children under 5 years of age ranged from 1.33 to 4.96 cases per 100 person-years, while the annual incidence of nosocomial infection ranged from 0 to 1.87 cases per 1000 days of hospitalization (0–68.2 per 100 person-years in hospital) [8]. The recent introduction of rotavirus vaccines reduced mortality, hospitalization rates, and the overall number of cases [9–11], but these effects were markedly higher in low-mortality countries since access to the vaccine in high-mortality countries is still too limited [1, 9]. Moreover, other factors such as differences in gut microbiome and human leukocyte antigen groups may play a role in reducing rotavirus vaccine efficacy in low-income countries [12]. There are currently four globally licensed live attenuated vaccines to be administered orally: Rotarix (a monovalent vaccine against the G1P antigen) and RotaTeq (a pentavalent vaccine against the proteins of G1, G2, G3, G4, and P serotypes) prevent about 82% and 60% of severe diarrhoea cases caused by rotavirus in developed and developing countries respectively [12, 13]. Rotavac (a monovalent vaccine against G9P antigen) and Rotasiil (a lyophilized pentavalent vaccine against proteins of G1, G2, G3, G4, and G9 serotypes) are most administered in Asia with similar results. As a whole, all the four vaccines are considered as highly effective in preventing severe rotavirus AGE [13].

In countries effectively immunizing children against rotavirus, norovirus is becoming the leading pathogen associated with medically attended AGE [14–17]. Bacteria account for 10–20% of AGE; *Shigella*, *Salmonella*, and enterotoxigenic and enteroinvasive *Escherichia coli* are the most common causative agents, and in low-to-middle income countries *Vibrio cholerae* is also quite common. Parasites, such as *Cryptosporidium*, *Giardia*, and *Entamoeba histolytica*, account for less than 5% of cases [1, 16, 18]. No etiologic surveillance system for infective gastroenteritis is currently active in our country, and a few studies have provided data on the prevalence of the different pathogens and on their correlations with clinical features in hospitalized children, confirming that viruses were the most common cause of severe AGE, with rotavirus having the main role [19, 20]. The aim of this single-centre study conducted in Pisa, Italy, was to analyse the clinical features, aetiology, and treatment in children admitted for acute gastroenteritis in 2019 and in 2012, focusing on the possible effects of the introduction of the rotavirus vaccine into the national vaccination plan since 2017, with a current rate of about 30% of vaccinated infants.

## Methods

### Study design and data collection

For this retrospective observational study, we reviewed the medical records of patients aged ≤ 18 years hospitalized for AGE (ICD-10 code A09.0) at the Paediatrics Unit, University Hospital of Pisa, from January to December 2019. Patients were excluded if they had persistent diarrhoea, chronic diarrhoea, eosinophilic gastroenteritis, cystic fibrosis, pancreatic insufficiency, inflammatory bowel diseases, coeliac disease, cow’s milk protein allergy, lactose intolerance, lymphocytic colitis, vomiting, or abdominal pain of uncertain aetiology. Demographical, clinical, diagnostic, and treatment data were recorded using a proforma. We collected data on the frequency and features of diarrhoea and vomiting, abdominal pain, fever, and hydration status by assessing skin turgor, as well as microbiological investigations: when stools were collected, the samples underwent stool culture to identify bacteria such as *Salmonella*, *Shigella*, *Campylobacter*, and *Yersinia* as well as immunoassay detection of rotavirus, norovirus, astrovirus, and adenovirus antigens and parasitology test. Routine blood tests, abdomen ultrasound, and data on oral or intravenous (IV) rehydration approaches and antiemetic administration were also recorded. The same data were collected in patients hospitalized for AGE from January to December 2012 for comparison with the results acquired in 2019. As for microbiological investigations, the same tests have been used both in 2012 and 2019, and in 2019 five patients underwent also molecular testing on the stools. Data relevant to the study were analysed and reported anonymously; thus, the ethical research committee approval was waived.

### Statistical analysis

The data were presented as number, percentage, mean ± SD, median, and interquartile range, when appropriate. Differences of categorical variables were analysed using the chi-squared test or, where the sample size was small, using Fisher’s exact test. Differences between means were determined using unpaired Student’s *T*-test and those between medians using the Mann–Whitney test, when appropriate. A *p*-value < 0.05 was considered significant. The analysis was carried out using the SPSS (Statistic Package for Social Science) software version 21.1 (IBM Corp., Armonk, NY).

## Results

### Demographic, clinical, microbiological, and treatment data of patients with AGE in 2019

**Fig. 1:**
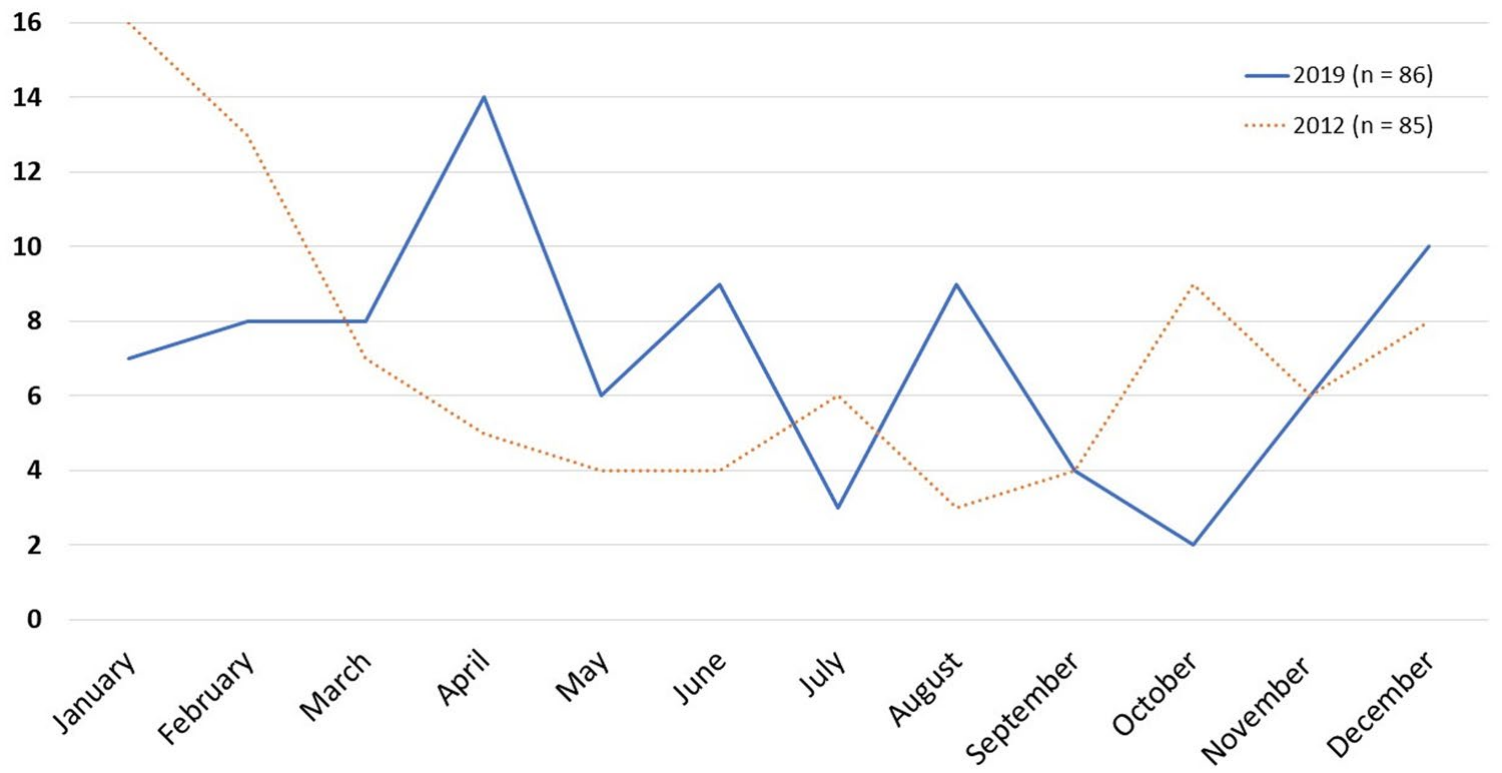
Trend of hospitalizations for acute gastroenteritis in Pisa University Hospital’s Pediatrics Unit in 2019

**Fig. 2:**
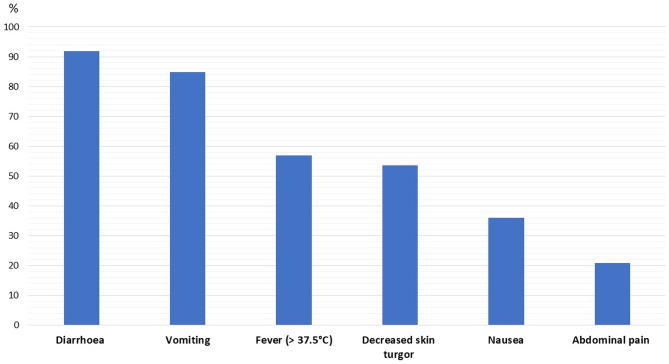
Prevalence of the main clinical manifestations in patients examined in 2019

**Fig. 3:**
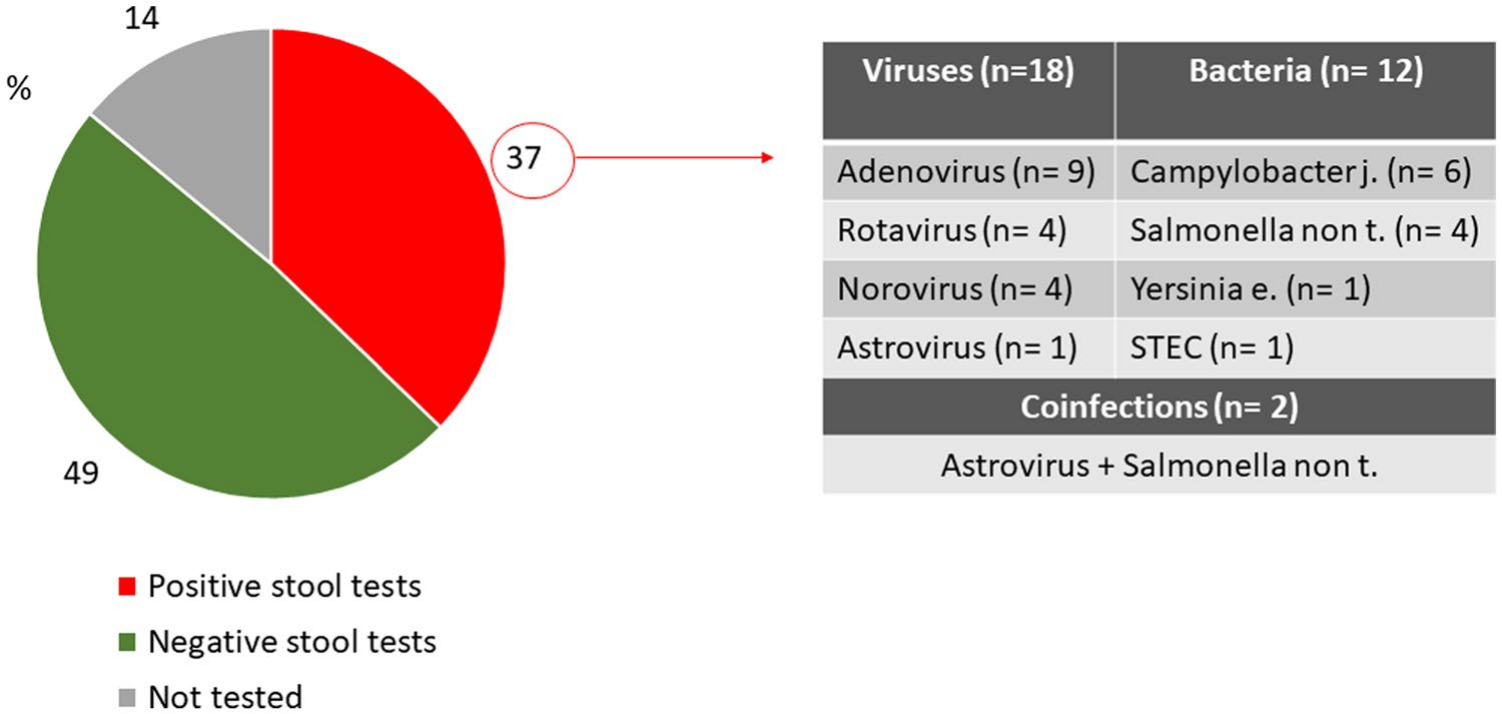
Results of stool testing in children hospitalized due to acute gastroenteritis in 2019

**Fig. 4:**
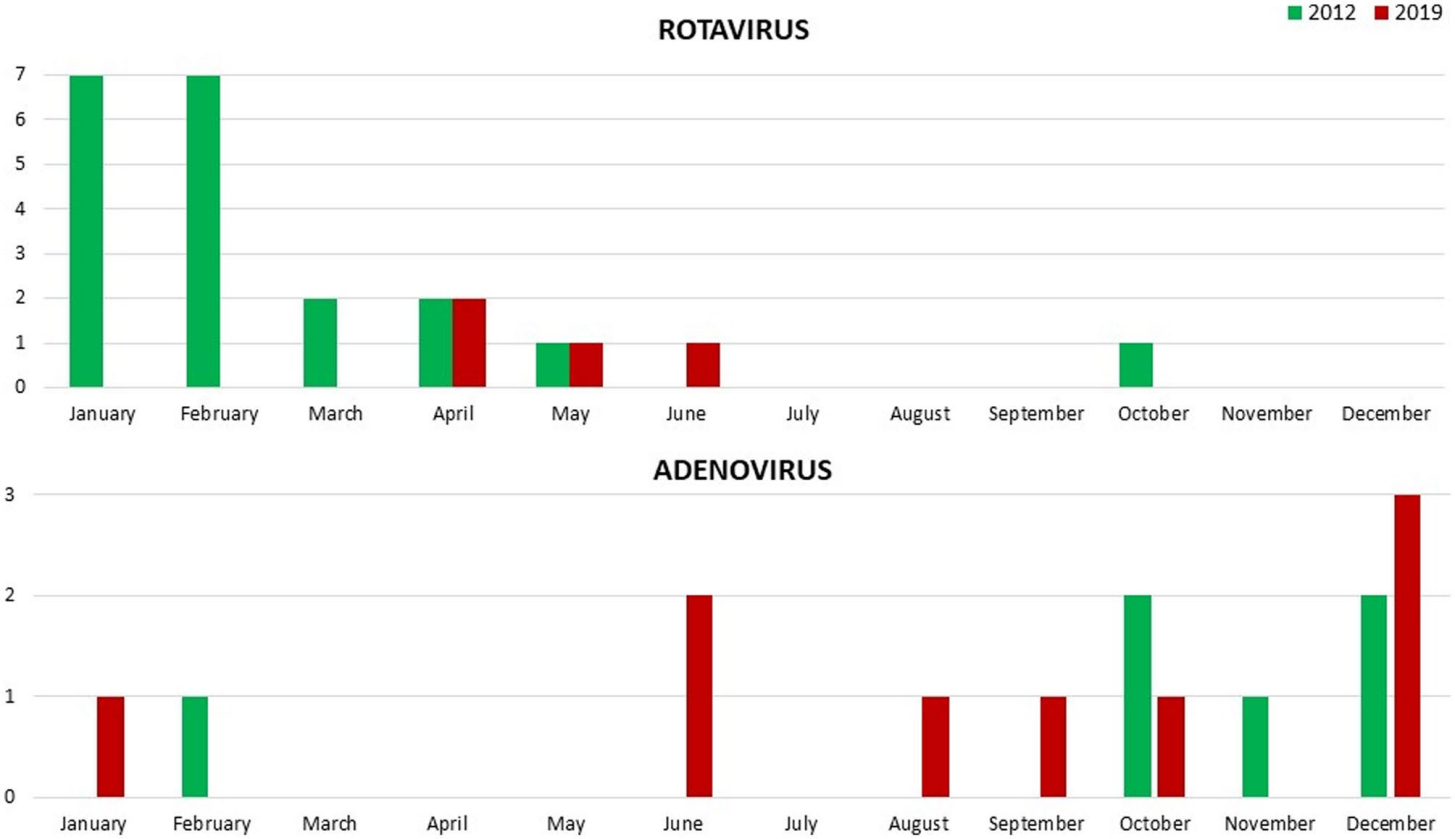
Number of hospitalizations caused by rotavirus or adenovirus gastroenteritis in 2012 and 2019

In 2019, 86 children aged 1 month to 18 years old (median 2.5 years [IQR 1.4–5.9]; 47 males, 55%), were discharged with a diagnosis of AGE; the majority of them were registered in April (15 cases) (Fig. 1). The most common symptoms were diarrhoea and vomiting (92% and 85%, respectively); fever, nausea, and abdominal pain were less common (Fig. 2). Muco-haematic diarrhoea was reported in two patients with *Campylobacter* infection. Decreased skin turgor was diagnosed in 54% of the study cohort. Stool samples were analysed in 74 out of 86 patients, detecting one or more pathogens in 32 cases (43%; 18 viral infections, 12 bacterial infections, and 2 viral-bacterial coinfections) (Fig. 3). One patient with Shiga toxin-producing *Escherichia Coli *(STEC) infection developed haemolytic uraemic syndrome; in this patient the diagnosis was obtained through a FilmArray Gastrointestinal multiplex PCR panel, while stool cultures later tested were negative. This molecular technique was performed in four other patients and confirmed the results obtained from traditional methods, even though multiple pathogens were identified in three of them, so a careful correlation with the clinical picture was useful to interpret those results correctly (Suppl. Table 1). None of the children with rotavirus AGE was vaccinated against this pathogen. Adenovirus infections were registered throughout the whole year, while rotavirus infections were registered almost exclusively in springtime (Fig. 4); norovirus and astrovirus infections occurred in the second half of the year.

The comparison between the two subgroups of patients with viral or bacterial AGE (excluding coinfections) revealed bacterial AGE was more common in older patients (8.1 ± 5.8 years and 3.2 ± 4.0 years, respectively; *p* = 0.02) and showed a higher frequency of abdominal pain (58% and 11%, respectively; p = 0.01), while a higher prevalence of vomiting (89% and 50%, respectively; *p* = 0.03) and nausea was found in viral AGE (50% and 8%, respectively; *p* = 0.02). No other difference was found (Table 1).

**Table 1:** Comparison of length of hospital stay, and signs and symptoms in bacterial and viral AGE in patients examined in 2019

	Bacterial AGE (*n* = 12)	Viral AGE (*n* = 18)	*p*
Age, years Range	8.1 ± 5.8 9 months–15.9 years	3.2 ± 4.0 3 months–17.8 years	0.02
Males, *n* (%)	6 (50)	14 (78)	n.s
Length of hospital stay, days	3.3 ± 1.1	3.6 ± 1.5	n.s
Diarrhoea, *n* (%)	11 (92)	18 (100)	n.s
Muco-haematic diarrhoea, *n* (%)	2 (17)	0	n.s
Vomiting, *n* (%)	6 (50)	16 (89)	0.03
Fever > 37.5 °C, *n* (%)	8 (67)	10 (56)	n.s
Nausea, *n* (%)	1 (8)	9 (50)	0.02
Abdominal pain, *n* (%)	7 (58)	2 (11)	0.01
Decreased skin turgor, *n* (%)	5 (42)	8 (44)	n.s
Blood sampling at admission, *n* (%) Increased WBC RCP > 0.5 mg/dL Increased ALT Increased creatinine	12 (100) 0 9 (75) 6 (50) 1 (8)	15 (83) 2 (13) 9 (60) 6 (40) 0	n.s n.s n.s n.s n.s

WBC: white blood cells; normal values: 5.000–20.000 in the third and fourth weeks of life, 6.000–17.000 up to 2 years of age, 5.000–15.000 in children aged 3 to 6 years, 4.500–13.5000 in patients aged 7 to 18 years. RCP: reactive-C-protein; normal values < 0.5 mg/dL. ALT: alanine transaminase; normal values: 5–25 U/L up to 2 years of age, 8–20 U/L in children and adolescents. Creatinine: normal values 0.6–1.2 in newborns, 0.2–0.4 up to 2 years of age, 0.5–1.0 in children and adolescents

Twenty-nine patients underwent abdomen ultrasound. This examination showed terminal ileitis in one patient with *Yersinia* infection, and mucosal wall thickening of the ileum and colon in three patients with *Campylobacter* infection. The other patients yielded unspecific findings (Suppl. Table 2). Blood examination was performed in 77 patients to assess inflammation markers and electrolytes; increase of inflammation markers and alteration of electrolytes were uncommon and overall mild. Twenty-three patients, of which three were with rotavirus infection, had a mild increase in alanine transaminase levels; 57% of them had dehydration by means of decreased skin turgor. IV rehydration alone was performed in 42% of patients, 29% received IV treatment after oral rehydration failure, 17% received oral rehydration alone, and 12% underwent gradual refeeding only. IV rehydration therapy included isotonic saline solution, Ringer’s lactate, saline solution combined with glucose solution, or a balanced paediatric crystalloid solution. Oral rehydration was carried out by hypotonic oral rehydration solution (ORS), as recommended by the European Society for Paediatric Gastroenterology Hepatology and Nutrition (ESPGHAN) [5]. Ondansetron was administered to 36 patients (42%).

### Comparison between 2019 and 2012 data

In 2012, 85 children aged 2 months to 17.5 years (median 2.3 years [IQR 1.3–5.1]) were admitted for the occurrence of AGE, with a maximum peak in January (16 patients, 49% males) (Fig. 1).

When comparing the data collected in the two study populations, a significant reduction in the mean length of hospital stay in 2019 was evident (2019: 3.1 ± 1.4 days; 2012: 3.7 ± 2.2; *p* = 0.03). Moreover, in 2019 we found a higher number of patients admitted with decreases skin turgor and fever in comparison with 2012 (54% and 34%, respectively, *p* = 0.01; 57% and 31%, respectively, *p* < 0.001) (Table 2). The number of patients that underwent microbiological stool tests did not differ between 2019 and 2012 (86% and 75%, respectively; *p* = 0.748), nor did the prevalence of bacterial AGE (16% and 13%, respectively; *p* = 0.382). Stools positive for viral antigens were significantly higher in 2012 than in 2019, with a higher number of rotavirus infections (67% and 22%, respectively; *p* = 0.003), most of which were in January and February (Fig. 4). Conversely, the number of AGE caused by adenovirus and the number of patients with negative stool testing were significantly higher in 2019 in comparison with the data of 2012 (50% and 10%, respectively, *p* = 0.002; 58% and 39%, respectively, *p* = 0.04). Ondansetron was not routinely administered in 2012, and no child in this study group received the drug, while it was administered in 36 children in 2019 (*p* < 0.001). The number of patients who received oral rehydration therapy alone in 2019 was higher than in 2012 (17% and 7%, respectively; *p* = 0.04). IV therapy alone was more frequently used in 2012 than in 2019 (66% and 42%, respectively; *p* = 0.002), while IV rehydration after oral rehydration failure was more commonly reported in 2019 than in 2012 (29% and 13%, respectively; *p* = 0.009).

**Table 2:** Comparison of clinical features, aetiology and treatment in patients admitted for AGE in 2019 and in 2012

	2019 (*n* = 86)	2012 (*n* = 85)	*p*
Age, years Range	Median 2.5 [IQR 1.4–5.9] 22 days–17.8 years	Median 2.3 [IQR 1.3–5.1] 2 months–17.5 years	n.s
Males, *n* (%)	47 (55)	42 (49)	n.s
Length of hospital stay, days	3.1 ± 1.4	3.7 ± 2.2	0.03
Diarrhoea, *n* (%)	79 (92)	72 (85)	n.s
Vomiting, *n* (%)	73 (85)	69 (81)	n.s
Fever (TC > 37.5 °C), *n* (%)	49 (57)	26 (31)	< 0.001
Nausea, *n* (%)	31 (36)	21 (25)	n.s
Abdominal pain, *n* (%)	18 (21)	22 (26)	n.s
Decreased skin turgor, *n* (%)	46 (54)	29 (34)	0.01
Microbiological investigations on stool samples, *n* (%)	74 (86)	64 (75)	n.s
- Viral infection Rotavirus Adenovirus Norovirus Astrovirus	18/74 (24) 4/18 (22) 9/18 (50) 4/18 (22) 1/18 (6)	30/64 (47) 20/30 (67) 3/30 (10) 6/30 (20) 1/30 (3)	0.005 0.003 0.002 n.s n.s
- Bacterial infection *Non-Typhi Salmonella* * Campylobacter jejuni* * Yersinia enterocolitica* * Escherichia coli*	12/74 (16) 4/12 (33) 6/12 (50) 1/12 (8) 1/12 (8)	8/64 (13) 5/8 (63) 3/8 (38) 0 0	n.s n.s n.s n.s n.s
- Viral-Bacterial coinfection	2/74 (3) Astrovirus and non-typhi Salmonella	1/64 (2) Rotavirus and non-typhi Salmonella	n.s
- Negative	42/74 (58)	25/64 (39)	0.04
IV fluid therapy alone, *n* (%)	36 (42)	56 (66)	0.002
IV fluid therapy after ORS failure, *n* (%)	25 (29)	11 (13)	0.009
ORS alone, *n* (%)	15 (17)	6 (7)	0.04
Gradual refeeding, *n* (%)	10 (12)	12 (14)	n.s
Length of IV fluid therapy, days	2.4 (1)	2.3 (1)	n.s

*IV* intravenous, *ORS* oral rehydration solution

## Discussion

AGE continues to cause a significant number of hospital admissions and outpatient evaluations in industrialized countries, especially in children under 5 years of age. In Italy, an average annual number of more than 40,000 hospitalizations for AGE in children under the age of 6 were recorded between 2005 and 2012 [21]. However, a specific surveillance system for AGE is not in place in Italy with the exception of foodborne AGE caused by pathogens such as *Salmonella* and STEC. Our study confirms data from other national studies showing that viral infections are the leading cause for admission in these children [19, 20]. However, we also found a significant decrease in rotavirus infections and an increase in adenovirus infections and in the number of patients with negative stool testing in 2019 when compared to 2012, when rotavirus vaccines were not available.

The overall median age of our patients was almost 2.5 years due to the higher prevalence of viral infections, which are more common in younger children [7]. Indeed, viral infections are typically acquired in community by oral-faecal transmission (higher risk settings are day cares), while bacterial infections are usually acquired from undercooked or contaminated food [22–24]. It should be also considered that viral intestinal infections usually induce a long-lasting immune response to specific viral strains so that those acquired during infancy are unlikely to recur [25]. In our study, clinical manifestations, both in viral and bacterial AGE, were comparable to the literature data, with diarrhoea being the most frequent symptom, followed by vomiting [4–6]. Vomiting and anorexia were the most common symptoms in viral AGE compared to bacterial ones. Abdominal pain frequently occurred during bacterial infections probably due to a more severe inflammatory enteritis compared to viral infections [24, 26]. As expected, we found that viral AGEs were most common in both 2019 and 2012. Among the patients examined in 2019, no pathogen was identified in 58% of patients. This result is in agreement with other studies suggesting that the common screening techniques cannot detect other pathogens which may have a higher prevalence than expected [27, 28]. Indeed, our patients were tested only for rotavirus, norovirus, astrovirus, and adenovirus, but not for other viruses, such as *Parechovirus*, *Aichivirus*, *Bocavirus*, and *Sapovirus*, which are beginning to have an increasingly frequent pathogenetic role in AGE since the introduction of the rotavirus vaccine [29, 30]. This is consistent with the fact that in 2012 the number of negative tests was substantially lower with a significantly higher number of positive tests for rotavirus compared to 2019. We found a significant decrease of rotavirus infections together with an increase of adenovirus infection, with the introduction of the rotavirus vaccine into the Italian national vaccination plan in 2017, according to other reports [2, 4, 28, 31–33]. We did not find any significant variation in norovirus and astrovirus infection prevalence, which in 2019 was similar to that reported in other European studies [34–36]. Although rotavirus vaccines lead to a decrease of rotavirus AGE, we did not observe a corresponding reduction in the number of hospitalizations, suggesting the emergence of other pathogens causing AGE. However, it should be noted that we found a mild reduction in length of stay in 2019 compared to 2012. In our study, *Campylobacter* and *non-typhi* *Salmonella* were the most common causes of bacterial AGE with no significant variation between 2012 and 2019, in agreement with European reports on bacterial AGE, which is typically acquired from contaminated food [5, 36, 37]. *Non-typhi* *Salmonella* is one of the leading causes of bacterial enterocolitis requiring hospitalization in children [37, 38] and potentially complicated by bacteraemia or sepsis in infants, immunocompromised patients, and in the elderly. Accordingly, in our study the only case of sepsis occurred in a 9-month-old child due to *Salmonella*. *Escherichia coli* infection was identified only in one patient with STEC complicated with haemolytic uraemic syndrome [38]; in this patient, microbiological diagnosis was achieved through molecular testing, while stool culture later resulted negative. As a whole, a microbiological diagnosis was not achieved in more than 60% of patients due to both the negative results at traditional testing and to the unavailability of stool samples. Together with limitations of traditional microbiological investigations, such as stool culture and immunoassays, this finding suggests the need of further studies on diagnostic techniques, especially on the role of molecular testing.

Other testing methods, such as blood examinations and abdominal ultrasound, are not recommended as routine tests in paediatric AGE, but they may be useful in patients with severe AGE with dehydration [5]. In our study, abdominal ultrasound and blood testing were performed in 34% and 90%, respectively. The high number of blood testing in our cohort of patients was related to the fact that about 70% of the patients received IV rehydration both alone and after oral rehydration failure, while ORS alone was administered only in 17% of patients. Although the ESPGHAN guidelines recommend ORS as the first step to treat mild-to-moderate dehydration [2, 5, 39–41], some studies showed that ORS is still infrequently applied as the first step of treatment in hospitalized children suffering from dehydration due to AGE [42]. Possible explanations behind this approach are as follows: the common feeling that oral rehydration takes longer than IV therapy to restore fluids, the belief that ORS is contraindicated in the case of vomiting, the poor palatability of available ORS, and parents’ or physicians’ preference for IV rehydration. These factors may also explain why we admitted about 50% of children showing no sign of severe dehydration. None of our patients received rehydration by nasogastric tube. This approach is recommended by international guidelines for mild-to-moderate dehydration in the case of failure of oral rehydration therapy before starting an IV [5], but its use is still limited worldwide probably because it is considered by both parents and physicians as more invasive than IV [43]. An important tool to promote oral rehydration is ondansetron administration, as a single dose leads to a substantial decrease of vomiting, enhancing the efficacy of oral rehydration therapy, thus reducing hospitalization and IV rehydration rate [44, 45]. Indeed, our data showed an increased use of ORS in 2019, both alone and followed by IV rehydration in the case of oral rehydration failure, together with a slightly reduced hospitalization length compared to 2012, when ondansetron was not routinely administered. Our study has some limitations. Firstly, as already mentioned, in 2019 stool testing was not performed in 14% of the patients, while 49% turned out negative, so that in more than 60% of the study population microbiological etiology was not defined. Secondly, our study included a small number of patients recruited only in two epidemic seasons; therefore, we could not exclude year fluctuations in pathogens. Moreover, the study was retrospective and data were retrieved from medical files. Since clinical information was sometimes reported partially, especially regarding presence of tears, refill time, and respiratory rate, it was not always possible to calculate dehydration scores, and we had to consider only decreased skin turgor as an index of dehydration. Another limit of our study is that we do not know the rate of rotavirus vaccination in the 2019 study group, since this information was reported in the medical files only for children with rotavirus infection (none of them had been vaccinated). Finally, our study included only hospitalized children, so that it cannot be considered representative of all cases of AGE, since most of these children are treated at home without performing any test.

## Conclusions

AGE often occurs as a mild and self-limited disease but represents a main cause of hospitalization in children under 5 years with significant costs for the health system. Despite the introduction of the rotavirus vaccine, viral infections are still the leading cause of AGE in hospitalized children. Our data suggest that new viral pathogens, such as adenovirus and other not-routinely screened viruses, are undergoing a selection process which is leading to their emergence as causative agents for AGE. ORS and ondansetron administration may be effective in reducing the number and length of hospitalizations.

## Supplementary Information

Below is the link to the electronic supplementary material.

Supplementary file1 (DOCX 16 KB)

Supplementary file2 (DOCX 15 KB)

## Data Availability

Data supporting the findings of this study are available from the corresponding author, Dr. Maria Di Cicco, upon reasonable request.
