# Supplementary material for: The evolving epidemiology of acute gastroenteritis in hospitalized children in Italy
Source: Eur J Pediatr. 2021 Jul 29;181(1):349–58. doi: 10.1007/s00431-021-04210-z (PMC8760218; doi:10.1007/s00431-021-04210-z)
Supplement: Supplementary file 1 — Supplementary file1 (DOCX 16 KB) [file 431_2021_4210_MOESM1_ESM.docx]

**Supplemental Table 1** – FilmArray Gastrointestinal multiplex PCR panel and other tests on the stool performance in 5 patients.

|  | **FilmArray** | **Immunoassay for viral antigens** | **Stool culture + *specific Yersinia, Campylobacter and Clostridium testing*** | **Parasitology test** | **Clinical manifestations** | **Microbiological**  **diagnosis at discharge** |
| --- | --- | --- | --- | --- | --- | --- |
| **Patient 1**  M, 1 yr | Clostridium difficile toxin, Giardia lamblia, Adenovirus | Adenovirus | Negative | Negative | Nausea, vomiting and diarrhoea | **Adenovirus** |
| **Patient 2**  M, 3 yrs | EPEC, STEC | Negative | Staphylococcus aureus | Negative | High fever, vomiting, diarrhoea, one episode of absence seizure and self-limiting HUS | **STEC** |
| **Patient 3**  F, 4.5 yrs | Salmonella, EPEC | Negative | Salmonella non typhi | Negative | High fever, vomiting, diarrhoea | **Salmonella non typhi** |
| **Patient 4**  M, 16 yrs | Yersinia, ETEC, STEC | Negative | Yersinia | Negative | High fever, diarrhoea, myalgia, abdominal pain, terminal ileitis at ultrasound | **Yersinia** |
| **Patient 5**  F, 16 yrs | Campylobacter | Negative | Campylobacter | Negative | Fever, diarrhoea, abdominal pain, ileocecitis at ultrasound | **Campylobacter** |
