# Supplementary material for: The evolving epidemiology of acute gastroenteritis in hospitalized children in Italy
Source: Eur J Pediatr. 2021 Jul 29;181(1):349–58. doi: 10.1007/s00431-021-04210-z (PMC8760218; doi:10.1007/s00431-021-04210-z)
Supplement: Supplementary file 2 — Supplementary file2 (DOCX 15 KB) [file 431_2021_4210_MOESM2_ESM.docx]

**Supplemental Table 2. –** Abdominal ultrasound findings in our study cohort (the test was performed on 29 children; 12 patients complained of abdominal pain).

|  | **Patients, n** |
| --- | --- |
| Increased air in the bowel | 12 |
| Mesenteric lymph node enlargement | 8 |
| Intestinal wall thickening  *Colon*  *Descending and sigma colon*  *Terminal ileum and ascending colon*  *Ileum*  *Stomach* | 8  2  2  2  1  1 |
| Free fluid between bowel loops or in Douglas pouch or pelvic region or iliac fossae | 8 |
| Gallstones | 2 |
| Normal findings | 4 |
